# Comparative transcriptome analysis of shoot and root tissue of *Bacopa monnieri* identifies potential genes related to triterpenoid saponin biosynthesis

**DOI:** 10.1186/s12864-017-3865-5

**Published:** 2017-06-28

**Authors:** Gajendra Singh Jeena, Shahnoor Fatima, Pragya Tripathi, Swati Upadhyay, Rakesh Kumar Shukla

**Affiliations:** Biotechnology Division, CSIR-Central Institute of Medicinal and Aromatic Plants (CSIR-CIMAP), Kukrail Picnic Spot Road, P.O. CIMAP, Lucknow, 226015 India

**Keywords:** *Bacopa monnieri*, Transcriptome, Transcription factors, Saponins, Secondary metabolites

## Abstract

**Background:**

*Bacopa monnieri* commonly known as Brahmi is utilized in Ayurveda to improve memory and many other human health benefits. Bacosides enriched standardized extract of *Bacopa monnieri* is being marketed as a memory enhancing agent. In spite of its well known pharmacological properties it is not much studied in terms of transcripts involved in biosynthetic pathway and its regulation that controls the secondary metabolic pathway in this plant. The aim of this study was to identify the potential transcripts and provide a framework of identified transcripts involved in bacosides production through transcriptome assembly.

**Results:**

We performed comparative transcriptome analysis of shoot and root tissue of *Bacopa monnieri* in two independent biological replicate and obtained 22.48 million and 22.0 million high quality processed reads in shoot and root respectively. After de novo assembly and quantitative assessment total 26,412 genes got annotated in root and 18,500 genes annotated in shoot sample. Quality of raw reads was determined by using SeqQC-V2.2. Assembled sequences were annotated using BLASTX against public database such as NR or UniProt. Searching against the KEGG pathway database indicated that 37,918 unigenes from root and 35,130 unigenes from shoot were mapped to 133 KEGG pathways. Based on the DGE data we found that most of the transcript related to CYP450s and UDP-glucosyltransferases were specifically upregulated in shoot tissue as compared to root tissue. Finally, we have selected 43 transcripts related to secondary metabolism including transcription factor families which are differentially expressed in shoot and root tissues were validated by qRT-PCR and their expression level were monitored after MeJA treatment and wounding for 1, 3 and 5 h.

**Conclusions:**

This study not only represents the first de novo transcriptome analysis of *Bacopa monnieri* but also provides information about the identification, expression and differential tissues specific distribution of transcripts related to triterpenoid sapogenin which is one of the most important pharmacologically active secondary metabolite present in *Bacopa monnieri*. The identified transcripts in this study will establish a foundation for future studies related to carrying out the metabolic engineering for increasing the bacosides biosynthesis and its regulation for human health benefits.

**Electronic supplementary material:**

The online version of this article (doi:10.1186/s12864-017-3865-5) contains supplementary material, which is available to authorized users.

## Background

*Bacopa monnieri* (L.) Wettst. Commonly known as “Brahmi” is a medicinal herb of great significance. It has been used worldwide due to its broad use of pharmaceutically important triterpenoid saponins mainly bacosides [1]. It is a perennial, creeping herb basically found in the wetlands of Australia, southern India, Europe, Asia, Africa, and South America. *Bacopa* is a genus of 70–100 aquatic plants of family Scrophulariaceae (Plantaginaceae) having succulent, oblong and 4–6 mm thick leaves arranged oppositely on the stem.

In the conventional system of Indian medicine, *Bacopa* is well treated as a drug to augment intelligence and memory function and combat the effects of mental stress [2]. It is also used for gastrointestinal infections, rejuvenation, skin disorders, epilepsy, pyrexia and analgesia [3]. Apart from that *Bacopa monnieri* extract is familiar to possess potent antioxidant and anticancer properties [4]. *Bacpa monnieri* has many efficacious compounds in addition to alkaloids, flavonoids, betulic acid, stigmasterol, beta-sitosterol and saponins (bacoside A, bacoside B, bacopasaponin C, bacopaside I, bacopaside II, bacopaside X, bacopaside N2). Due to its affluent active components, the plant has been largely utilized as a nootropic digestive attention and for improving respiratory functions [5]. Earlier studies also revealed that bacosides from *Bacopa monnieri* leaf extract is used to treat memory functions and impairment [6]. Evaluation and extraction of bacosides from areal part of the plant using HPLC chromatographic technique have been performed and reported that highest concentration of bacoside A is present in stolon (9.54 mg/g dry wt) followed by leaves (4.73 mg/g dry wt) and roots [7].

In bacteria and higher eukaryotes MVA pathway is one of the important metabolic pathways. The product of MEP and MVA pathway such as 3,3-dimethylallyl diphosphate (DMAPP) and isopentenyl diphosphate (IPP) act as the important mediator in several secondary metabolites production in plants [8, 9]. Several enzymes take part in structural alteration of these intermediates by undergoing substitution, oxidation, and glycosylation producing various glycosylated triterpenoids. Squalene synthase which is an important governing step in sterol and triterpenoid biosynthesis catalyzes the conversion of farnesyl pyrophosphate (FPP) molecules to squalene. Squalene epoxidase is involved in the epoxidation of squalene to form 2,3-oxidosqualene which is the branch point in the biosynthesis of steroidal and terpenoid sapogenin. Cyclization of 2,3-oxidosqualene catalyzed by a class of oxidosqualene cyclases (OSCs) to form distinct secondary metabolite backbones. Later cytochrome P450-dependent monooxygenases (CYP450) and glycosyltransferases (GTs) govern oxidation, hydroxylation, and glycosylation step to yield triterpenoid saponins and sterols with distinct structures and biological activities [10].

Emerging high-throughput sequencing technologies has changed the pace of DNA sequencing in plants and animals which perform comprehensive profiling of RNA expression [11, 12] other than model plant organisms were constrained molecular genetics applications have been implemented. RNA sequencing administers complete transcriptome expression profiles of selected plant cells or tissues, thereby allowing novel approaches to analyze functional genomics, in addition more precise measurement of transcript level and their isoforms than other methods. Transcriptome assay using NGS sequencing has been extensively used to identify genes encoding enzymes ramified in distinct steps of biosynthetic pathways in medicinal plants. Some include the description of genes encoding enzyme catalyzed metabolic steps mediated in the biosynthetic pathway of artemisinin in *Artemisia annua* [13, 14], withanolides in *Withania somnifera* [15, 16], cannabinoids in *Cannabis sativa* [17], ginsenosides in *Panax ginseng* [18], glycyrrhizin in *Glycyrrhiza uralensis* [19], picrosides in *Picrorhiza kurrooa* [20], cardiac glycoside in *Calotropis procera* [21], menthol content in *Mentha* species [22], medecinal diterpene in *Andrographis paniculata* [23] and biosynthesis of steroidal saponins in *Asparagus racemosus* [24].

As the synthesis and accumulation of specialized plant metabolites in different tissues depends on the age of the plant and is also highly influenced by the different developmental stages. It was reported earlier that in *P. ginseng* and *P. quinquefolius*, leaves accumulate higher content of triterpenoids during earlier growth stages whereas older plants have a higher content of triterpenoids in root [25]. Comparative transcriptome analysis including root and shoot tissues to identify transcripts involved in saponin biosynthesis is already reported for many plant species like *Asparagus racemosus* [24], *Panax notoginseng* [26] *Gynostemma pentaphyllum* [27]. The maximum bacoside content was identified in stolon followed by leaf and usually the aerial portion extract is used for checking its efficacy in memory impairment responses. So in this study comparative shoot versus root paired-end transcriptome sequencing was performed for *Bacopa monnieri* to identify genes involved in triterpenoid saponin. Efforts were also made to identify potential transcription factors, CYP450s and UGTs that play a key role in regulation and diversification of secondary metabolites. Paired-end sequencing strategy extends the mapped fragment length to 200–500 bp so it is expected to be useful in obtaining longer reads, allows for detecting alternate splice junctions, deletions, insertions, and is useful for de novo transcriptome assembly [28]. Transcripts generated after assembly were functionally elucidated in different gene ontology, some of the secondary metabolic pathway related transcripts were selected for further validation. The fold change induction of differentially expressed transcripts in the shoot and root tissue was evaluated using DGE (Digital gene expression) profile, followed by q-RT PCR analysis. The transcriptome data generated in this study will provide new insight into the enzyme transcripts involved in the secondary metabolic pathway of this important medicinal plant.

## Results

### Transcriptome sequencing and de novo assembly

To characterize the transcriptome of *Bacopa monnieri*, we sequenced cDNA libraries prepared from the shoot and root tissues of *Bacopa monnieri* using Illumina HI Seq 2000 System yielding an entire 24.85 million and 24.31 million raw reads. The Illumina paired-end raw reads were quality checked using FastQC. Total 22.48 and 22.0 million reads were generated after sequencing of the shoot and root tissues of *Bacopa monnieri* subsequently after removing adapter containing low quality reads. Maximum and minimum read length was 101 and 50 bases. Total 78.10% reads of the shoot and 79.53% reads of root samples were aligned to their respective assemblies. A total of 26,412 genes got annotated in root sample while 18,500 genes annotated in the shoot sample. The quality of raw reads was determined by using SeqQC-V2.2. Short read values were assembled by using assembly software which improves the quality of transcriptome assembly. Elementary fragmented assembly generated by using Velvet assembler programme which was further corrected by Oases with the help of dynamic and static filters. In root sample 61.9 million reads and in shoot sample 45.3 million reads were used for assembly by velvet programme whereas by Oases program a total of 45.5 million reads in root sample and 29.9 million reads in shoot sample were assembled. A systematic approach for Illumina transcriptome was summarized in Additional file 1. Velvet and Oases program generates 89,367 and 72,916 contigs from shoot having hash length 53 while 121,814 and 91,807 contigs from the root having hash length were 57 respectively. We obtained higher average contig length by using Oases programme (1329.1 ± 1352.9 in root tissue and 1467.1 ± 1366.8 in shoot tissue) as compared with Velvet programme (478.4 ± 398.9 in root tissue and 479.1 ± 388.3 in shoot tissue). Transcriptome sequencing was performed using two independent biological replicates along with their significant *P* values. Overall summary of Hi-Seq Illumina transcriptome assembly obtained after biological replicate is mentioned in Table 1. In both samples, the average length of contigs ranges from 300 bases to more than 1000 bases (Additional file 2). Final sequences obtained from both the tissues were submitted to the SRA database of NCBI with accession number SAMN04216962.

**Table 1:** Summary of HI-Seq Illumina Transcriptome assembly of *Bacopa monnieri* root and shoot tissue after two independent biological replicate

	Unigenes_Shoot**	Unigenes_Root**
Number of transcripts identified	1,42,295	1,78,308
Maximum Contig Length	16,771	16,744
Minimum Contig Length	300	300
Average Contig Length	999.7 ± 826.2	931.5 ± 793.6
Median Contig Length	751	1116
Total Contigs Length	14,22,49,012	16,60,98,010
Total Number of Non-ATGC Characters	0	0
Percentage of Non-ATGC Characters	0	0
Contigs > = 100 bp	1,42,295	1,78,308
Contigs > = 200 bp	1,42,295	1,78,308
Contigs > = 500 bp	93,697	1,11,010
Contigs > = 1 Kbp	49,751	54,765
Contigs > = 10 Kbp	16	16
N50 value	1407	1281

Double astrick symbol indicates the representative transcrips of shoot and root

### Functional characterization and GO analysis

**Fig. 1:**
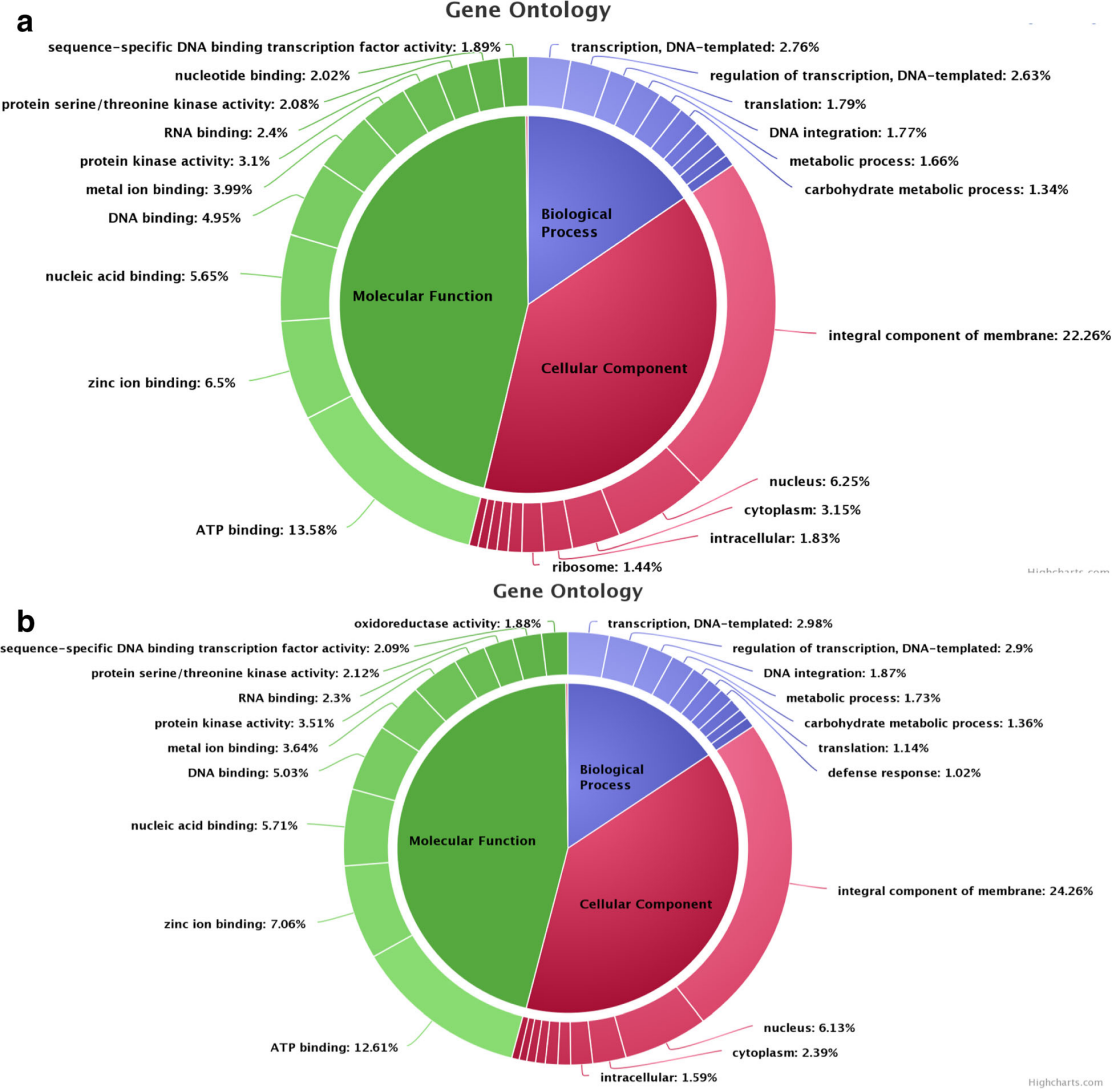
Top ten most represented GO terms in each of the three GO domains. Depending on NR elucidation, Gene Ontology classification of assembled Unigenes was functionally annotated and categorized into three main GO domain i.e. biological processes, cellular components, and molecular function in both (**a**) Root and (**b**) Shoot tissue. Data represented in pi-chart is drawn after two independent biological replicates (*n* = 2)

The functional annotation for *Bacopa monnieri* assembled sequences was based on sequence similarity searches against public databases such as UniProt, KEGG and NCBI Nr using the basic local alignment search tool (BLASTx) algorithm with an E-value cutoff of <10–10. Out of the total 92,649 annotated sequences, 722 (0.77%) were showing close homology with *Arabidopsis* while 2936 unigenes were complementary with order Lamiales. A high fraction of Unigenes (67.55%) was found complementary to both *Arabidopsis* and Lamiales out of which 26,394 (28.48%) unigenes were unannotated (Additional file 3). Depending on NR elucidation, Gene Ontology (GO) classification has been used to distinguish the available functions of the unigenes. At least one GO term annotation was strongly assigned to all the unigenes. At least 47% and 52% of the transcripts in root and shoot respectively were functionally annotated at high confidence value (e1–5). Functionally annotated transcripts were then categorized into three main GO domain i.e. biological processes, cellular components, and molecular function (Additional file 4). In root, the best represented groups of biological processes were transcription (2.76%), regulation of transcription (2.63%) and translation (1.79%) while in cellular component, the unique sequences related to the integral component of membrane (22.26%), nucleus (6.25%) and cytoplasm (3.25%) were well-represented categories (Fig. 1a). The transcripts belong to the major subgroups of molecular function category included ATP binding (13.58%), zinc ion binding (6.50%) and nucleic acid binding (5.65%). In shoot, for biological process, a large number of genes were annotated to transcription (2.98%), regulation of transcription (2.90%) and DNA integration (1.87%) while for cellular component category; the leading three categories were integral component of membrane (24.26%), nucleus (6.13%) and cytoplasm (2.39%). A high percentage of the unique sequences in root were annotated to ATP binding (12.61%), zinc ion binding (7.06%) and nucleic acid binding (5.71%) in the molecular function category (Fig. 1b). These GO annotations provide an extensive knowledge on assembled transcript belonging to different functional categories of *Bacopa monnieri.*

### Mapping and enrichment of KEGG pathway

The Kyoto Encyclopedia of Genes and Genomes (KEGG) pathway provides better insight about pathway maps revealing the knowledge on the molecular interaction and reaction networks. To better understand about biological pathways operating in *Bacopa monnieri*, a BLASTx search against the KEGG protein database was performed on the assembled unigenes with an E-value threshold of 10–5. Pathway analysis was done by using KAAS Server. *Arabidopsis thaliana* were considered as reference organisms for pathway identification. Among 85,579 annotated unigenes in root and 74,624 unigenes in the shoot, 37,918 and 35,130 unigenes were mapped to 133 KEGG pathways in root and shoot respectively. Based upon the number of unigenes assigned, carbohydrate metabolism, translation, lipid metabolism, amino acid metabolism and signal transduction are the dominant metabolic pathways operating in shoot and root. Among secondary metabolic pathways, the most represented category was phenylpropanoid biosynthesis (365 unigenes), terpenoid backbone biosynthesis (340 unigenes), carotenoid biosynthesis (125 unigenes), diterpenoid biosynthesis (77 unigenes) and zeatin biosynthesis (57 unigenes) in root followed by terpenoid backbone biosynthesis (358 unigenes), phenylpropanoid biosynthesis (286 unigenes), carotenoid biosynthesis (126 unigenes), diterpenoid biosynthesis (52 unigenes) and zeatin biosynthesis (51 unigenes) in shoot.

### Differential expression analysis of unigenes

The expression level of the unigenes in root and shoot tissue of *Bacopa monnieri* was calculated using RPKM method (Reads Per kb per Million reads). The average log10 RPKM values for root and shoot tissue were found to be 2.4821 and 2.1286 respectively which limits from <1.0 to >8.0 (Additional file 5). Master transcripts representing root and shoot tissue were generated by clustering through CD-HIT. Fold change induction along with their RPKM values which manifest that a maximum number of transcripts are expressed with a fold change induction ranging between 2 to 4 fold (Additional file 6). Differential gene expression was depicted by calculating the RPKM ratio of each gene in two different samples obtained after two independent biological replicates. Information about DGE and its statistical analysis is mentioned in Table 2. From the DGE data, we found that 246,584 unigenes were expressed differentially in which 40,055 unigenes were clearly expressed in root tissue whereas 5531 unigenes were clearly expressed in shoot tissue. Total 188,802 unigenes were found to be expressed in both shoot and root tissues. Using BLASTx analysis of different transcripts involved in secondary metabolism having significantly preferred expression in the shoot and root tissues of *Bacopa monnieri* revealed that most of them were functionally involved in terpenoid and phenylpropanoid pathway in which we found that 36 unigenes in the shoot and 51 unigenes in root were involved in triterpenoid biosynthesis.

**Table 2:** Summary of DGE data obtained after two independent biological replicate and its statistical analysis

Total Unigenes available		2,46,584		
No. of unigenes in each category for Root and Shoot samples	Total	Up	Down	Neutral
No. of unigenes expressed in both samples	188,802	36,262	33,556	118,984
No. of unigenes expressed only in Root sample	40,055	NA	NA	NA
No. of unigenes expressed only in Shoot sample	5531	NA	NA	NA
No. of P-significant unigenes	10,871	6951	3920	0
No. of Q-significant unigenes	3659	2345	1314	0
Software used for carrying out DGE	DESeq			
Sample taken as control for DGE	Root			
Sample taken as Treated for DGE	Shoot			

### Mevalonic acid pathway and transcripts related to Saponin biosynthesis

Transcripts involved in mevalonic acid pathway were found to be specifically upregulated in the root tissues. Under control conditions, we found that most of the transcripts involved in the first phase of triterpenoid biosynthesis were upregulated in root as compared to shoot. Acetyl CoA C-acetyltransferase showed the highest expression followed by Hydroxymethylgutryl CoA reductase which is the rate limiting step in mevalonic acid pathway. In *Bacopa monnieri* transcriptome various transcripts encoding enzymes related to steroidal and triterpenoid sapogenin biosynthesis pathway were identified and their differential gene expression was monitored after two independent biological replicate along with their mean values (Table 3). Most of the transcripts related to terpenoid and steroidal biosynthesis pathway in the data were found in shoot tissue as compared to root tissue. Unique annotated sequences that are responsible for sapogenin backbone biosynthesis were first sequestered, except for methylesterol monooxygenase, we found the putative transcripts that encode all the enzymes for steroidal sapogenin biosynthesis. Transcriptomic study of *Bacopa monnieri* reveals the putative transcripts related to cytoplasmic MVA pathway which includes includes Hydroxymethylgutryl CoA reductase (96 unigenes, EC 1.1.1.34), Mevalonate kinase (48 unigenes, EC 2.7.1.36), Phosphomevalonate kinase (24 unigenes, EC 2.7.4.2), Diphosphomevalonate decarboxylase (36 unigenes, EC 4.1.1.33), Geranylgeranyl diphosphate synthase (60 unigenes, EC 2.5.1.1 2.5.1.10 2.5.1.29), Farnesyl diphosphate synthase (60 unigenes, EC 2.5.1.12.5.1.10), squalene monooxygenase (36 unigenes EC 1.14.13.132), cycloartenol synthase (12 unigenes EC 5.4.99.8), cycloeucalenol cycloisomerase (5 unigenes EC 5.5.1.9), Delta 14 sterol reductase (4 unigenes EC 1.3.1.70), cholestenol delta isomerase (6 unigenes EC 5.3.3.5) and β-glucosidase (6 unigenes EC 3.2.1.21) which were specifically found in shoot tissue while some of the transcripts such as Acetyl CoA C-acetyltransferase (84 unigenes, EC 2.3.1.9), Hydroxymethylgutryl CoA synthase (120 unigenes, EC 2.3.3.10), Squalene synthase (36 unigenes, EC 2.5.1.21), Sterol 24-C-methyltransferase (6 unigenes, EC 2.1.1.41), Lathosterol oxidase (6 unigenes, EC 1.14.21.6), 7 dehydrocholesterol reductase (6 unigenes, EC 1.3.1.21) and delta 24-sterol reductase (5 unigenes, EC 1.3.1.72) were specifically found in root tissue. Transcripts which are specifically involved in triterpenoid sapogenin biosynthesis at later modification steps includes UDP-glucosyltransferase (5 unigenes, EC 2.4.1), β-amyrin synthase (5 unigenes, EC 5.4.99.39) and Cytochrome P450 (12 unigenes, EC 1.14).

**Table 3:** List of transcripts found in DGE data related to Steroidal and Terpenoid sapogenins biosynthesis obtained after two independent biological replicates along with their mean values

Transcript ID	Transcript annotation	Total Unigenes	Expression value (Root)	Expression value (shoot)
Root_c66568_g1_i2	Acetyl-CoA C acetyltransferase [EC:2.3.1.9]	84	2372.0404	816.1341
Root_c116346_g1_i2	Hydroxymethyl gutryl CoA synthase [EC:2.3.3.10]	120	166.7105	65.9895
Shoot_c28002_g1_i1	Hydroxymethyl gutryl CoA reductase [EC:1.1.1.34]	96	1355.5136	409.38630
Shoot_c81148_g2_i2	Mevalonate kinase [EC:2.7.1.36]	48	45.8906	35.9789
Shoot_c54668_g1_i2	Phosphomevalonate kinase [EC: 2.7.4.2]	24	43.8436	20.8782
Shoot_c83433_g2_i1	Diphosphomevalonate decarboxylase [EC: 4.1.1.33]	36	122.5076	39.7524
Shoot_c79849_g1_i2	Geranylgeranyl diphosphate synthase [EC:2.5.1.1]	60	321.8642	1168.3271
Shoot_c91138_g2_i6	Isopentenyl pyrophosphate [EC:5.3.3.2]	58	153.1148	99.9740
Shoot_c50773_g1_i1	Farnesyl diphosphate synthase [EC:2.5.1.1.2.5.1.10]	60	82.8082	77.3196
Root_c69154_g3_i1	Squalene synthase [EC:2.5.1.21]	36	384.5765	769.8759
Shoot_c54028_g1_i1	Squalene monooxygenase [EC:1.14.14.7]	36	4.4532	43.5067
Shoot_c57924_g1_i1	Cycloartenol synthase [EC:5.4.99.8]	12	16.7469	36.0105
Root_c111034_g6_i3	Sterol 24-methyltransferase [EC:2.1.1.41]	6	11.2460	26.9001
Shoot_c1673_g1_i1	Cycloeucalenol cycloisomerase [EC:5.5.1.9]	5	0.8680	4.0081
Shoot_c49436_g1_i2	Obtusifoliol 14α demethylase [EC:1.3.1.70]	4	1297.0030	1756.1878
Shoot_c44076_g1_i1	Cholestenol delta isomerise [EC:5.3.3.5]	6	166.1151	224.9541
Shoot_c58739_g1_i2	Beta-amyrin 28 oxidase [EC:1.14.13.201]	1	63.9678	90.9996
Shoot_c88943_g1_i2	Cytochrome P450 [EC:1.14.]	169	1132.6685	207.2123
Shoot_c90073_g1_i5	1-deoxy-D-xylulose 5-phosphate reductoisomerase [EC:1.1.1.267]	4	142.0968	397.2787
Shoot_c93177_g1_i1	1-deoxy-D-xylulose-5-phosphate synthase [EC:2.2.1.7]	2	947.3682	1510.9590

### Study of metabolic pathway genes

**Fig. 2:**
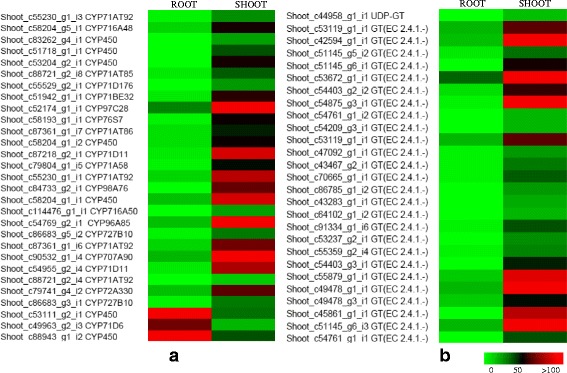
Heat map representing the tissue-specific expression of CYP450 and UGTs. Differential expression of CYP450 (**a**) and GTs (**b**) that were upregulated in shoot tissue as compared to root as the control. The fold change expression data represented here is obtained after two biological replicates with *P* ≥ 0.05 (significant). Colour scale representing normalized fold induction is shown in the figure

Triterpenoid Saponins (bacoside A, bacoside B, bacopasaponin C, bacopaside I, bacopaside II, bacopaside X, bacopaside N2) present in *Bacopa monnieri* are the prime source of its important medicinal properties and are formed by cytoplasmic MVA and plastid MEP pathways. Analyzing transcriptome data we found that 36 transcripts in the shoot and 51 transcripts in root are involved in triterpenoid and sesquiterpenoid biosynthesis. Earlier studies demonstrated that elementary reactions of isoprenoid biosynthesis take place in leaf tissue and further modification, storage of saponins were supposed to occur in the root tissue, therefore, a higher amount of saponins were accumulated in roots [29, 30]. Tissue-specific transcriptome analysis of *Bacopa monnieri* suggest the distribution of enzyme transcript involved in saponin biosynthesis and its precursors present in both the tissue. Based upon expression analysis using transcriptome sequencing their is the possibility of biosynthesis and further modifications such as glycosylation and oxidation using UGTs and cytochrome-P450 occurs in leaf. Although the initial enzymes involved in precursor biosynthesis are also present in root tissue which suggest the involvement of both the tissue in the metabolic pathway. The metabolic analysis however, has suggested it is the aerial part which mostly contains higher bacoside content and utilized at most of the places for its pharmacological activity. Real-time expression analysis shows that the expression of β-amyrin synthase gene (OSC) is higher in root tissue as compared to shoot and was also upregulated after MeJA treatment and wounding. The downstream pathway of triterpenoid saponins involves a range of modifications including oxidation and addition of sugar moiety to saponin backbone. In our transcriptome data, 194 unigenes of the CYP450s were found in root and 169 unigenes were found in shoot tissues. In addition to this 117 unigenes of UGT in root and 95 unigenes of UGTs in the shoot were reported. Among the DEGs, 29 CyP450s and 27 UGTs were found to be upregulated in the shoot as compared to root tissue (Fig. 2a and b).

### Putative transcripts involved in Phenylpropanoid biosynthesis pathway

Most of the known transcripts associated with the phenylpropanoid and monolignoid pathway were found to be clearly expressing in root in comparison with shoot tissue of *Bacopa monnieri*. In our transcriptome data, we found that 286 unigenes in the shoot and 365 unigenes in root tissue were specifically involved in phenylpropanoid biosynthetic pathway. Chalcone synthase (24 unigenes, EC 2.3.1.74) which is an important primary enzyme in the phenylpropanoid pathway was found to be highly expressed in root tissue leads to the production of various secondary metabolites being flavonoids, isoflavonoids, and anthocyanins. The expression level of cinnamoyl alcohol dehydrogenase (CAD) was detected and was found upregulated in root tissue which is assumed to be involved in monolignoid biosynthesis.

### Transcription factor families

**Fig. 3:**
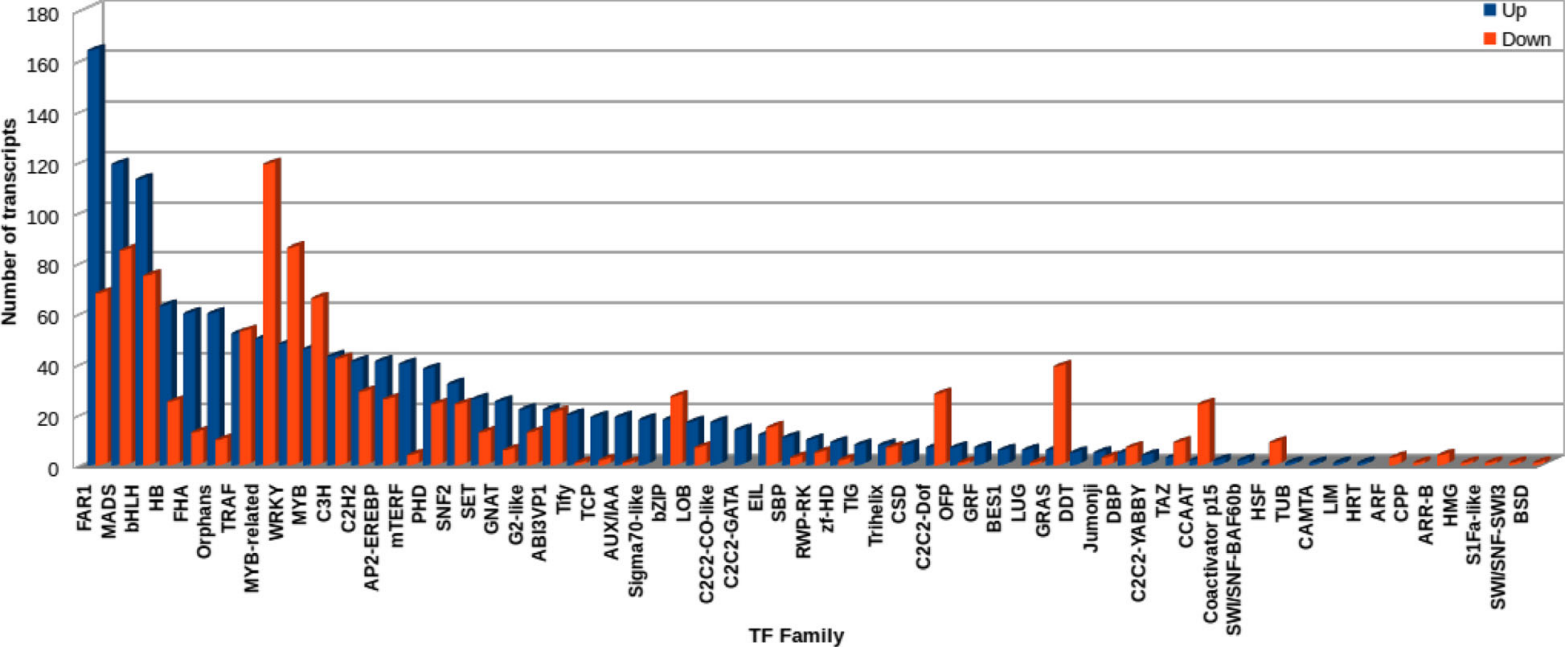
Transcription factors analysis. Differential expression profile of *Bacopa monnieri* transcripts related to different transcription factor families in shoot tissue as compared with control root tissue obtained after two independent biological replicates (*n* = 2). FAR1 and MADS box superfamily of transcription factor was found to highly upregulated in the shoot as compared to root tissue. The fold expression data represented here is obtained after two biological replicates with *P* ≥ 0.05 (significant)

Transcription factors are the class of protein which bind to the promoter regions of a downstream gene and modulate its expression at different levels. Relative modulation of different plant metabolic processes reveals the involvement of different transcription factors for coordinated regulation of gene expression. The Transcription factors with an RPKM value of more than or equal to 2 were selected for the differential expression analysis. By comparing their sequences with known transcription factor gene families, a total of 33,559 and 42,622 annotated transcripts were identified in the shoot and root samples which are involved in transcription, including DGEs. These transcription factors were distributed to 81 families including some transcription factor gene families like MYB, MADS, NAC, basic Helix-Loop-Helix (bHLH), AREBP, WRKY and much more. In root, members of C3H (8.11%) transcription factor family were found to be most abundant followed by FAR1 (5.81%) and MADS box (4.84%) superfamily while in shoot, FAR1 (6.99%) was found to be most abundant followed by C3H (6.71%) and MADS box (5.39%). DGE of transcription factors and their upregulation or downregulation in shoot tissues as compared with control root tissue obtained after two independent biological replicates with significant mean values showed that the maximum number of transcripts upregulated in shoot belongs to FAR1, MADS box and bHLH superfamily (Fig. 3).

### Functionally annotated transcripts validation by using qRT-PCR analysis

**Fig. 4:**
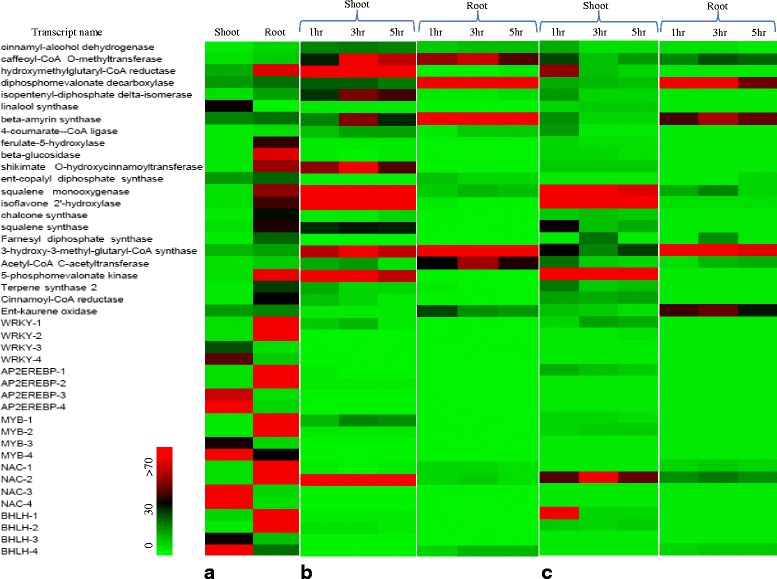
Heat map showing tissue-specific expression of transcripts in the shoot and root tissue. **a** Validation of up-regulated transcripts and tissue specific expression of transcription factors in shoot and root by using qRT-PCR analysis. **b** After MeJA treatment. **c** After wounding. All these transcripts were related to secondary metabolite biosynthesis. Actin and ubiquitin were taken as the internal reference gene and the relative abundance of each transcript in roots and shoot tissue was compared. The data represents three independent biological (*n* = 3) and experimental replicates performed with standard deviations. *Colour scale* representing normalized fold induction is shown in the figure

To corroborate that the annotated transcripts from computational and sequence analysis were certainly expressed and also to evaluate the differential gene expression profile between shoot and root tissues, 43 tissue specific transcripts (both root and shoot) cognate to secondary metabolite biosynthesis were selected for qRT-PCR analysis (Additional file 7A). Some transcription factors from the shoot and root tissues that belong to AREBP, MYB, NAC, bHLH and WRKY superfamily were selected for validation by using qRT-PCR (Additional file 7B). Under control conditions, we found that most of the transcripts related to triterpenoid and phenylpropanoid pathway were highly expressed in root as compared to shoot (Fig. 4a). After validation, it was found that some transcripts related to secondary metabolic pathway were highly expressed like 5 phosphomevalonate kinases (58 fold), Hydroxymethylglutaryl-CoA reductase (57 fold), Squalene monooxygenase (47 fold), Isoflavon 2′ hydroxylase (37 fold) and Squalene synthase (33 fold). AREBP, MYB, NAC, bHLH and WRKY (1, 2 are root specific) transcription factor families were found to be specifically upregulated in root tissues while AREBP, MYB, NAC, bHLH and WRKY (3, 4 are shoot specific) were specifically upregulated in shoot tissues. Out of above 23 transcripts Hydroxymethylglutaryl-CoA reductase, 5 phosphomevalonate kinases, Squalene synthase and Squalene monooxygenase are involved in triterpenoid and steroid sapogenin biosynthesis through cytoplasmic Mevalonate kinase pathway. Triterpenoids and various other secondary metabolites in several plant species are derived mainly from cholesterol but less knowledge is available about monolignoid and steroidal sapogenin biosynthetic pathways. In our transcriptome, two or more unigenes were assigned to the same enzyme, the reason behind that those unigenes may be the fragment of a single transcript or different members of the same gene family.

### Analysis of secondary metabolic pathway related transcripts by quantitative real-time PCR (q RT-PCR) after wounding and methyl jasmonate (MeJA) treatment

MeJA acts as an important elicitor in a wide spectrum of signaling pathways and also regulates the activity of distinct enzymes involved in the secondary metabolic pathways. MeJA has been proved to elicit the production of several compounds such as alkaloids, terpenoid and phenolic phytoalexins, coumarins, and taxanes in many plant species [31]. Wounding is a common damage that occurs to plants as a result of abiotic and biotic stress. It is hypothesized that plants may have evolved several mechanisms in response to wounding. We have selected 43 transcripts including transcription factor families from shoot and root related to secondary metabolite biosynthesis specifically to triterpenoid and phenylpropanoid pathway and also monitor their relative expression after 1, 3 and 5 h of exogenous application of MeJA treatment and wounding in tissues respectively. All the transcripts and transcription factor families showed increased accumulation after 3 h of MeJA treatment (Additional file 8A, B). In *Arabidopsis*, MeJA was found to activate both the general and downstream aspects of the phenylpropanoid biosynthesis pathway [32]. To be steady with *Arabidopsis*, the expression of most phenylpropanoid biosynthesis genes in *Bacopa monnieri* was upregulated by MeJA in different degree (Fig. 4b).

**Fig. 5:**
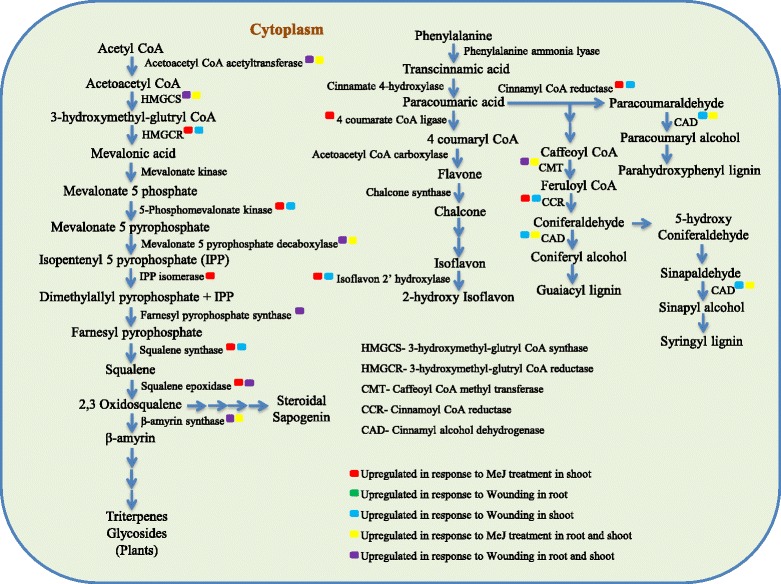
Representation of secondary metabolic pathway genes and tissue specific expression in response to MeJA and wounding in *Bacopa monnieri*. Systematic representation of the cytoplasmic mevalonic acid pathway, phenylpropanoid and monolignoid biosynthesis pathway genes. Enzymes were highlighted and marked according to their tissue specific expression in the shoot and root tissue along with their up-regulation after exogenous MeJA application and Wounding in *Bacopa monnieri*. The relative expression of selected transcripts were represented in comparison to actin and ubiquitin taken as an internal control

Wounding in shoot tissue showed early expression of transcripts while the differential accumulation of transcripts at different time points in root was observed (Fig. 4c and additional file 9). Acetyl CoA acetyltransferase, 3-hydroxymethylglutryl CoA synthase, Mevalonate 5-pyrophosphate decarboxylase and β-amyrin synthase of mevalonic acid pathway and Cinnamoyl alcohol dehydrogenase, caffeoyl CoA methyltransferase of monolignoid biosynthesis pathway showed induced accumulation in both shoot and root tissue after MeJA treatment (Fig. 4b). Acetyl-CoA acetyltransferase, 3-hydroxymethylglutryl CoA synthase, Mevalonate 5-pyrophosphate decarboxylase, Squalene epoxidase, Farnesyl pyrophosphate synthase and β-amyrin synthase of the mevalonic acid pathway and caffeoyl CoA methyltransferase of monolignoid biosynthesis pathway showed induced accumulation in both shoot and root tissue after wounding (Fig. 4c). Squalene monooxygenase, Isoflavone 2-hydroxylase, and NAC-2 transcription factor family showed enhanced accumulation of transcripts around 50 folds in shoot tissue after MeJA treatment and wounding. Besides mevalonate kinase, rest of the transcripts of cytoplasmic mevalonic acid pathway showed increased accumulation in root only. The selected transcripts which are involved in the secondary metabolic pathway and their tissue specific expression in response to exogenous MeJA treatment and wounding are shown in Fig. 5.

## Discussion

*B. monnieri* is an important medicinal plant with the number of pharmacological uses. Although it is pharmacologically important but the genome or transcriptome information is very confined. In NCBI only 68 ESTs, 68 nucleotide sequences and 35 protein sequences are accessible from *Bacopa monnieri*. The present manuscript has studied the comparative de novo transcriptome analysis of shoot and root tissue of *Bacopa monnieri*. The identified transcript from shoot and root will further be useful to understand the biosynthetic pathway of pharmacologically useful secondary metabolite present in different tissues of *B. monnieri.*

De novo comparative shoot and root transcriptome of *Bacopa monnieri* generates more than 44.68 million high-quality reads which were further used for assembly of data. The mean length of unigenes in the resulting assembly was more than 1000 bp which was found to be longer than the result showed in earlier studies [33, 34]. We reflect the pace of high-quality sequencing by bringing about more than 100% high-quality bases for both shoot and root tissues. Assembly process could be hampered by the presence of low-quality reads including adapters resulting in misassemblies or degraded contigs. Consequently, low-quality bases including adapter sequences were removed before continuing with further assembly. Our results proposed that the de novo transcriptome assembly from *Bacopa monnieri* were assembled correctly, which was further monitored by the high fraction of unigenes coordinated with known proteins along with higher N50 value.

The best match for individual unigene search against the NCBI non-redundant database and KEGG was promoted to assign functional GO annotation under biological process, cellular component groups and molecular function. The abundance of varied GO assignments to unigenes featured the assortment of genes possibly represented in *Bacopa monnieri* shoot and root transcriptome data. Unigenes were mapped onto KEGG, we had found that several unigenes are involved in distinct secondary metabolites biosynthesis. Unigenes without the match are most likely related to untranslated regions, noncoding RNA and short sequence protein without any domain or assembly mistakes. Moreover, the sequences encoding a large range of transcripts characterized under the cellular component category which points out the requirement of various transcripts for maintenance of the cellular structure. In support of the annotation, it reveals that the transcripts encoding a large number of enzymes involved in triterpenoid backbone biosynthesis specifically occur in roots.

Bacosides are the main constituent of *Bacopa monnieri* that was chemically a triterpenoid saponin and the pharmacological properties are mainly attributed to these triterpenoid saponin compounds present in the plant extract [2]. Three new dammarane-type triterpenoid saponins i.e. bacopasaponins A, B and C alongwith pseudojujubogenin (bacopasaponin D) were identified by spectroscopic and chemical transformation methods [35]. In our transcriptome, we have found transcripts related to both steroidal and terpenoid saponin which suggests the presence of both types of saponins in this plant. Transcripts related to bacosides biosynthesis like β amyrin synthase, CYP450 monooxygenases, GTs predominates in root tissue as compared to shoot but expression analysis of differentially expressed transcripts related to CYP450 and UGTs showed that they are highly expressed in the shoot as compared to root tissue. This implies that later stage modification of saponin backbone that involves several hydroxylation and glycosylation steps takes place in shoot tissue. In addition to saponin, several other transcripts involved in Phenylpropanoid biosynthesis were also found in our transcriptome data which suggests the presence of some flavonoids, lignin and phenylpropanoids compounds that was not reported earlier in *Bacopa monnieri*.

MeJA acts as an elicitor and plays critical roles in various plants metabolic processes by enhancing the expression of genes associated with secondary metabolic pathways. On the other hand wounding generally, induces the pathways genes related to secondary metabolites involved in defense response [36]. Transcript of Cinnamyl alcohol dehydrogenase (CAD) from *Bacopa monnieri* was showing induced expression in response to MeJA and wounding in shoot tissue which is involved in monolignoid biosynthesis*.* Similarly wounding in *Silanum elaeagnifolium* showed significant increased stress response Caryophyllene, Geranyl linalool and two terpenes transcripts [37].

Our result showed differential expression of transcripts involved in secondary metabolism in both root and shoot of *Bacopa monnieri*. Transcripts of Cinnamoyl CoA dehydrogenase, Caffeoyl-CoA methyltransferase, HMG-CoA reductase, Squalene monooxygenase and Isoflavone 2-hydroxylase of *Bacopa monnieri* showed induced expression after MeJA treatment in shoot specific manner. In root caffeoyl-CoA-O-methyltransferase, diphospho mevalonate decarboxylase, β-amyrin synthase, HMG- CoA-synthase showed induced expression in a root-specific manner. Identified and selected transcripts related to saponin and other secondary metabolic pathway related transcripts were showing better response towards MeJA treatment in shoot tissues. The increased expression of an important enzyme of triterpenoid backbone biosynthesis after MeJA treatment in shoots such as HMGCR, 5-Phospho mevalonate kinase, Mevalonate 5-pyrophosphate decarboxylase, Isopentenyl 5-pyrophosphate isomerase, Squalene synthase and Squalene epoxidase further showed that primary reactions of isoprenoid backbone biosynthesis take place in the shoot. The enhanced expression of β-amyrin synthase in response to MeJA and wounding in root tissue of *Bacopa monnieri* which is an important enzyme involved in triterpenoid sapogenin biosynthesis at later stages further reveals that triterpenoid saponin biosynthesis might be enhanced when the plant recognizes certain elicitor under stressed conditions. Similar stimulatory effects of MeJA on the biosynthetic pathway of other triterpenoid saponin compounds have already been reported [38]. It was also showed that triterpenoid biosynthetic enzyme β-amyrin synthase increased up to 50-fold by introducing MeJA to cell suspension cultures of *M. truncatula* [39]. Illumina paired-end RNA-seq for de novo reconstruction of *P. minus* leaf transcriptome to identify differentially expressed genes in response to MeJA elicitation [40].

Transcription factors interact with the promoter region of the downstream gene and regulate its expression. These are the site of single step manipulation in the complex metabolic pathway as they can regulate simultaneously a number of steps in a pathway. During transcriptome analysis, we have identified the specific transcription factors which are expressing in either shoot or root. The real-time expression analysis of these transcription factors in response to MeJA and wounding has further identified NAC and bHLH transcription factor showing the better response in comparison with other families such as WRKY and AP2/ERF protein. Similarly, dynamic changes in the expression pattern were observed in different transcription factor families in *Populus hopeiensis* after ABA treatment [41]. Also, the transcriptome based tissue-specific expression of transcription factor genes provides important information for understanding the development and transcriptional regulation of the paper mulberry [42]. The transcriptional regulatory responses of these transcripts after MeJA treatment and wounding would be useful further to identify transcripts responsive to elicitation. The above information can be utilized as an effective component through genetic engineering for enhancing the metabolite content.

## Conclusion

In the present study, the comparative transcriptome analysis of shoot and root tissues of *Bacopa monnieri* was performed to know about the putative transcripts involved in the secondary metabolite biosynthetic pathway of an important medicinal herb. The differential expression pattern of pathway genes and transcription factors in the shoot and root tissues of *Bacopa monnieri* suggest tissue-specific synthesis, accumulation followed by modification of these metabolites might occur by different signaling cascades. This study provides a useful resource of biosynthetic pathway transcripts identified from this important Ayurvedic medicinal plant with human health benefits. The identified transcripts will pave the foundation to metabolically engineer this plant with pharmacologically increased metabolite content.

## Methods

### Sampling and RNA extraction

For transcriptome analysis wild type *Bacopa monnieri* (CIM-JAGRITI) plants were collected from the green house of CSIR-CIMAP (Lucknow). Samples of the shoot and root tissues were collected from the 1 month old plant and immediately chilled in liquid nitrogen and stored at −80 °C. Total RNA from approximately 100 mg of frozen tissue of both shoot and root was extracted using TRIzol (Invitrogen) following the manufacturer’s recommendations.

### cDNA library preparation and sequencing

Illumina multiplexed Sequencing mRNA library preparation was carried out at Genotypic Technology’s Genomics Facility using Sure-select strand-specific RNA library preparation kit following manufacture’s protocol (Cat # 5500–0116). Briefly, the mRNA was fragmented for 4 min at elevated temperature (94oC) in the presence of divalent cations and first strand cDNA is synthesized. Strand specificity is maintained by the addition of actinomycin D. Second strand cDNA was synthesized using second strand synthesis mix. The cDNA was cleaned by AgencourtAmpure XP SPRI beads (Beckman-Coulter). After end repair and addition of “A” base, adapters were ligated to the cDNA molecules. After ligation SPRI cleanup was performed. The library was amplified and indexed using 10 cycles of PCR for improvement of adapter-ligated fragments. Finally, the concentration and size of the cDNA library were determined with a Qubit 2.0 Fluorometer (Invitrogen, Carlsbad, CA, US) and an Agilent 2100 Bioanalyzer (Agilent Technologies, Santa Clara, CA, USA).

### De novo assembly and bioinformatics analysis of sequences

After quality determination, sequencing was performed by using paired-end Illumina Hi-sequencing 2000. Quality control programme i.e. SeqQC-V2.2 was used to determine the aspects of sequence reads. Reads with adaptor and vector contamination was discarded. Finally, the reads with more than 20% Q < 20 bases were also removed. The high quality concatenated reads of shoots and roots were used to develop de novo assembly by De Bruijn graph algorithm based software tools. Reads were massed into contigs at distinct k-mer value using Velvet (https://www.ebi.ac.uk/~zerbino/velvet/). After these resulting contigs were merged into transcript isoforms using Oases_v0.2.08 (https://www.ebi.ac.uk/~zerbino/oases/), which was specially designed for the de novo assembly of transcripts using short reads. Different hash lengths were used to select the best de novo assembly. Construction of transcript isoforms was done by using paired-end information. Different specifications of assembly such as N50 length, average contig length, the number of contigs were upgraded to retrieve the best-assembled data with high coverage length. Combined transcripts from the multiple k-mer assemblies were run through the CD-HIT (CD-HIT v4.5.4 http://www.bioinformatics.org/cd-hit/) program to remove redundant transcripts sharing 100% identity.

### Transcriptome annotation

The putative function of unigenes obtained in transcriptome was subjected to BLASTx analysis against the non-redundant protein sequences (nr) of PDB, UniProt, PIR, all annotated protein sequences of *Arabidopsis* (available at the *Arabidopsis* information resource) and *Oryza sativa*. The results of only the best hit were extracted and the hits with an E-value <1E-05 were considered to be significant. Uniprot, Swiss-Prot, and TrEmBL databases were utilized for homology search. Gene ontology (GO) annotations were made by using Blast2GO program that was based on Nr annotations of NCBI and unigenes were categorized into three different GO terms i.e. molecular function, cellular component, and biological process. After assigning Enzyme Commission numbers (ECs) to unigenes from Fast Annotator, KEGG (Kyoto Encyclopedia of Genes and Genomes) was used to determine metabolic pathways related with them. Bidirectional best hit information was achieved, related to the metabolic pathway with the help of KAAS (KEGG Automatic Annotation Server http://www.genome.jp/tools/kaas/) by using reference database of model plant *Arabidopsis thaliana* (thale cress) and *Oryza sativa* japonica (Japanese rice).

### Identification and analysis of differentially expressed genes (DEGs)

The definite algorithm was developed to identify genes differentially transcribed in the shoot and root tissue of *Bacopa monnieri*. Both leaf and root samples were combined and clustered using CD-hit. Reads of both the samples were used against unigenes using Bowtie-2 to obtained read count profile. DESeq was used to identify the differential gene expression. RPKM values were adjusted with RNA and total read number to normalize transcript molar concentration and length. To compare the RPKM of DEGs we use false discovery rate (FDR), a statistical method to test the correction for comparisons. We obtained the *p*-value using the differential gene expression test. FDR adjustment was used to determine the *p*-value threshold in multiple tests and analyses. When a single gene is represented by two or more unigenes, the longest unigenes among them was selected to check the relative expression level and its coverage. For genes with missing values in a specific sample, the RPKMs were adjusted to 0.001. To identify differentially expressed genes, more precise criteria, with smaller FDR and bigger fold-change values, were used. Functional correlation can be observed within genes with similar expression patterns. For each of these analyses, a *P* value <0.05 was required for differences to be considered statistically significant.

### Transcription factor analysis

Transcripts coding for transcription factors in the transcriptome of *Bacopa monnieri* shoot and root tissues were anticipated by searching against all transcription factor protein sequences present in PlantTFcat (http://plantgrn.noble.org/PlantTFcat/about.gy) using BLASTX with an E-value cutoff 1e^−6^ and domain analysis within transcription factor was performed by using InterPro.

### Quantitative real-time PCR (qRT-PCR) analysis

Twenty-three transcripts and 20 transcription factors which are either highly up-regulated or tissue-specific in shoot and root with inherent aspects in secondary metabolite biosynthesis were selected for validation by qRT-PCR (list of primers for qRT-PCR analysis is given in Additional file 10: Table S1). For control sample, 2-month-old seedlings of *Bacopa monnieri* plants were harvested, shoot and root tissues were separated, quickly wiped, frozen in liquid nitrogen and stored at −80 °C for further use. Total RNA was isolated from the shoot and root tissue separately by GITC method. To study the effect of MeJA and wounding on the expression pattern of randomly selected 23 potential transcripts related to secondary metabolite biosynthesis, and 20 transcripts related to transcription factors were selected. Green house grown 1-month-old seedlings of *Bacopa monnieri* were used for MeJA treatment and wounding. MeJA (250 μM) solution in DMSO and Triton-X was prepared and used for spraying over aerial parts of healthy plants. After spraying the solution the seedlings were covered by perforated transparent plastic bags to maintain proper transpiration. Wounding treatment was given to the plantlets by puncturing the whole plant on leaves and stem. Samples of MeJA and Wound treated plants were collected after 1, 3 and 5 h of treatment separately and washed with MQ to remove soil and other contaminants. Total RNA from the shoot and root samples were extracted separately by GITC method. The RNA samples were dissolved in MQ and examined using the NanoDrop ND-8000. cDNA synthesis was done by using 2 μg of RNA from the shoot and root tissues of treated as well as control plant with high capacity cDNA Reverse Transcription Kit (Applied Biosystems). Diluted cDNA samples were utilized as a template for qRT-PCR analysis. The reaction was carried out on a 7500 FAST Real-Time PCR System (Applied Biosystems, USA) using the RealMasterMix (SYBR Green, Takara). Actin and ubiquitin were used as endogenous control for normalization of expression levels of unigenes. Relative gene expression levels were calculated using the 2^-ΔΔCt^.

## Additional files

Additional file 1: Flow diagram representing shoot versus root HI-seq Illumina transcriptome sequencing, data analysis and annotation of *Bacopa monnieri* unigene obtained. (DOCX 100 kb)

Additional file 2: Overview of the *Bacopa monnieri* Hi-Seq Illumina transcriptome sequencing and assembly. Length distribution of the transcripts obtained from de novo assembly of contigs after clustering in both (A) Root and (B) Shoot tissues. The length of high quality transcripts varies between <300 to >1000 bp. The data represented here is a mean value of two independent biological replicate. (DOCX 108 kb)

Additional file 3: Venn diagram for number of unigenes showing sequence homology with *Arabidopsis* and order Lamiales. The diagram shows the overlapping unigenes in the *Arabidopsis* and order Lamiales. A total 62,590 unigenes were found in both tissues, while some unigenes only were found in specific tissues. (DOCX 35 kb)

Additional file 4: Gene Ontology (GO) classification of the *Bacopa monnieri* HI-seq Illumina transcriptome. GO term assignments to *Bacopa monnieri* unigenes based on significant plant species hits against the NR database. Results are summarized into three main GO categories (biological process, cellular component, molecular function) and 30 sub categories. (DOCX 99 kb)

Additional file 5: Scattered plot showing the distribution and expression of transcripts among the uigenes obtained after two independent biological replicate (*n* = 2). DGEs for every gene family transcripts obtained in shoot and root tissue showing their up regulation. The data represented scattered plot is having a p significant value of *p* ≤ 0.05. (DOCX 257 kb)

Additional file 6: Fold change expression of transcripts in shoot tissue in comparison with root tissue. Differential gene expression was depicted by calculating the RPKMs ratio of each gene in two different independent biological replicates where samples (*n* = 2). The figure represents the fold change values corresponding to their number of transcripts. Fold change induction along with their RPKM values which shows that maximum number of transcripts are expressed with a fold change induction ranging between 2 to 4 fold for both upregulated and downregulated transcripts. The data represents the mean value of two independent biological replicates with *P* ≥ 0.05 (significant). (DOCX 75 kb)

Additional file 7: (A) Validation of selected 23 up regulated transcripts in shoot and root by using qRT-PCR analysis. (B) Tissue specific expression of selected transcription factors by using qRT-PCR analysis. All these transcripts were related to secondary metabolite biosynthesis. Actin and ubiquitin was used as the internal reference gene and the relative abundance of each gene transcript in roots and shoot tissue was compared. The data represents three independent biological and experimental replicates performed with standard deviations. (DOCX 69 kb)

Additional file 8: Relative expression levels of secondary metabolism related gene transcripts (A) and Transcription factor families (B) in *Bacopa monnieri* shoot and root tissues after 1, 3 and 5 h of MeJA treatment. Total RNA was extracted from root and shoot of 1 month old plant and expression level of identified transcript was analyzed by q-RT PCR. Actin and ubiquitin was used as the internal reference gene and the relative abundance of each gene transcript in root and shoot tissue was compared. The data represents three independent biological and experimental replicates performed with standard deviations. (DOCX 269 kb)

Additional file 9: Relative expression levels of secondary metabolism related gene transcripts (**A**) and Transcription factor families (**B**) in *Bacopa monnieri* shoot and root tissues after 1, 3 and 5 h of Wounding. Total RNA was extracted from root and shoot of 1 month old plant and expression level of identified transcript was analyzed by q-RT PCR. Actin and ubiquitin was used as the internal reference gene and the relative abundance of each gene transcript in root and shoot tissue was compared. The data represents three independent biological and experimental replicates performed with standard deviations. (DOCX 140 kb)

Additional file 10: Table S1. List of the primers used for selected genes to check their expression using RT-PCR. Parameters used in the velvet and oases assembly. (DOC 125 kb)
