# Supplementary figures and images for: Comparative transcriptome analysis of shoot and root tissue of Bacopa monnieri identifies potential genes related to triterpenoid saponin biosynthesis
Source: BMC Genomics. 2017 Jun 28;18:490. doi: 10.1186/s12864-017-3865-5 (PMC5490213; doi:10.1186/s12864-017-3865-5)

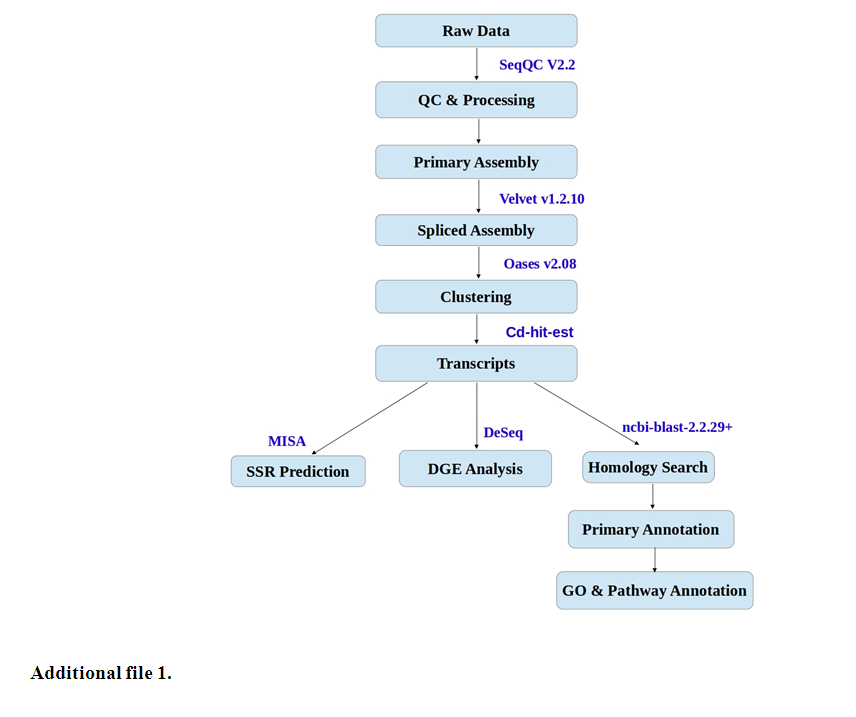

Supplement: Supplementary file 1 — Flow diagram representing shoot versus root HI-seq Illumina transcriptome sequencing, data analysis and annotation of Bacopa monnieri unigene obtained. (DOCX 100 kb) [file 12864_2017_3865_MOESM1_ESM.docx]

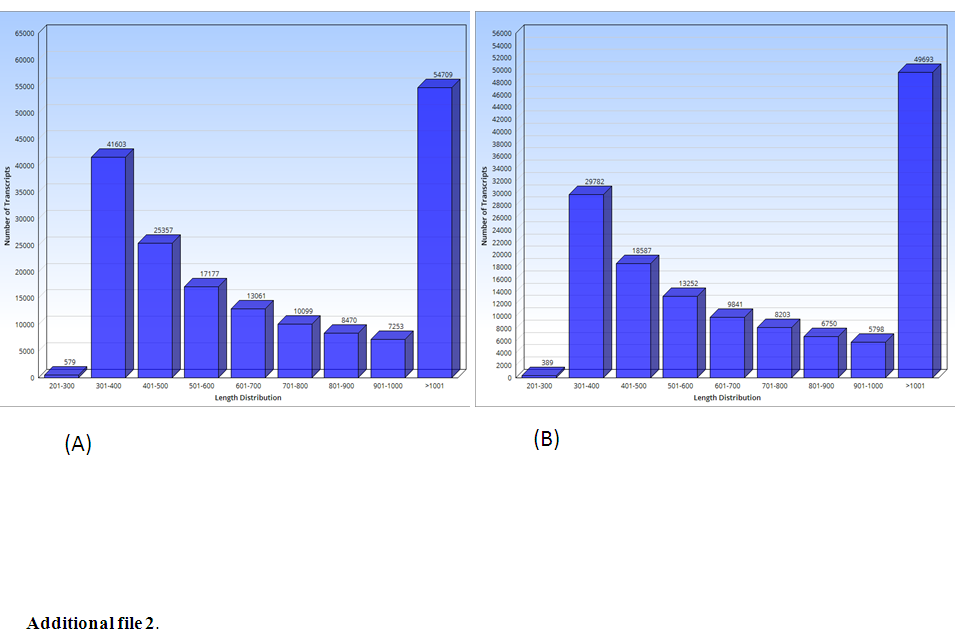

Supplement: Supplementary file 2 — Overview of the Bacopa monnieri Hi-Seq Illumina transcriptome sequencing and assembly. Length distribution of the transcripts obtained from de novo assembly of contigs after clustering in both (A) Root and (B) Shoot tissues. The length of high quality transcripts varies between <300 to >1000 bp. The data represented here is a mean value of two independent biological replicate. (DOCX 108 kb) [file 12864_2017_3865_MOESM2_ESM.docx]

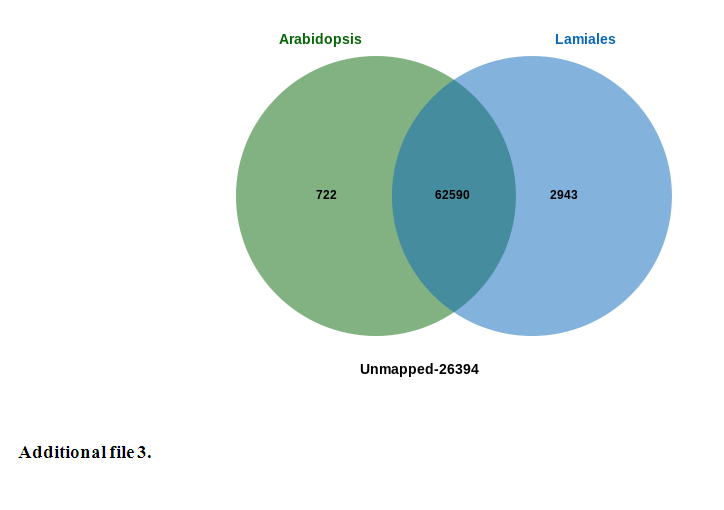

Supplement: Supplementary file 3 — Venn diagram for number of unigenes showing sequence homology with Arabidopsis and order Lamiales. The diagram shows the overlapping unigenes in the Arabidopsis and order Lamiales. A total 62,590 unigenes were found in both tissues, while some unigenes only were found in specific tissues. (DOCX 35 kb) [file 12864_2017_3865_MOESM3_ESM.docx]

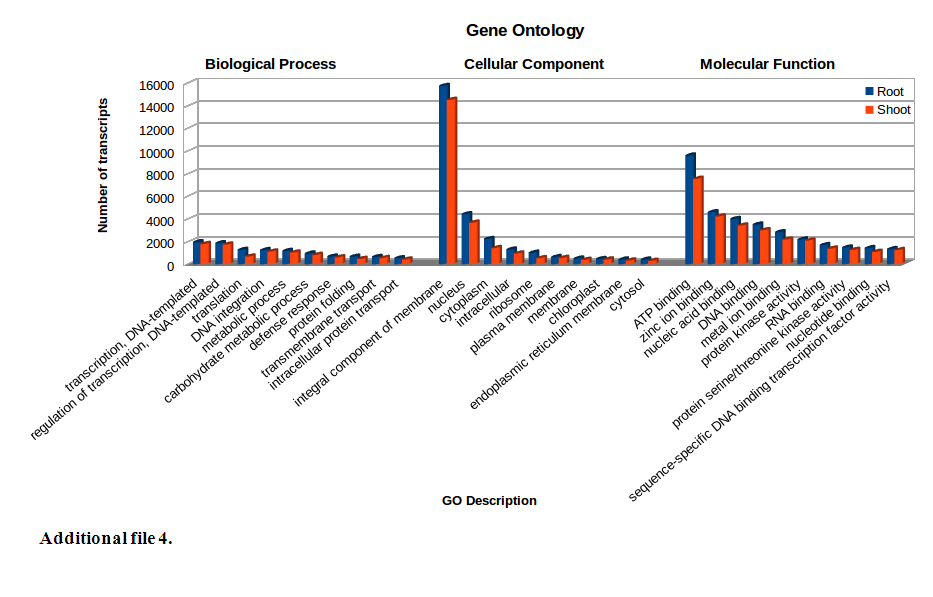

Supplement: Supplementary file 4 — Gene Ontology (GO) classification of the Bacopa monnieri HI-seq Illumina transcriptome. GO term assignments to Bacopa monnieri unigenes based on significant plant species hits against the NR database. Results are summarized into three main GO categories (biological process, cellular component, molecular function) and 30 sub categories. (DOCX 99 kb) [file 12864_2017_3865_MOESM4_ESM.docx]

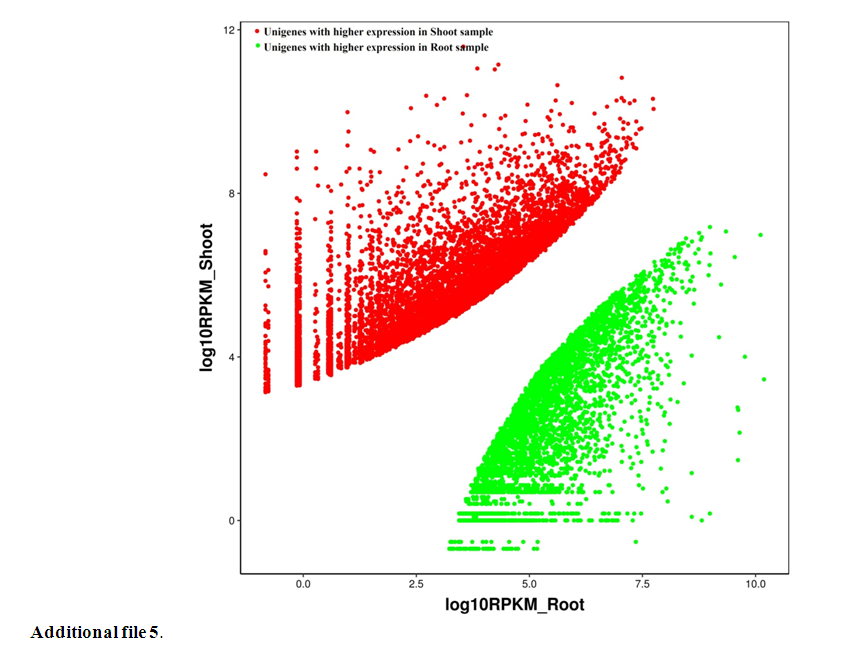

Supplement: Supplementary file 5 — Scattered plot showing the distribution and expression of transcripts among the uigenes obtained after two independent biological replicate (n = 2). DGEs for every gene family transcripts obtained in shoot and root tissue showing their up regulation. The data represented scattered plot is having a p significant value of p ≤ 0.05. (DOCX 257 kb) [file 12864_2017_3865_MOESM5_ESM.docx]

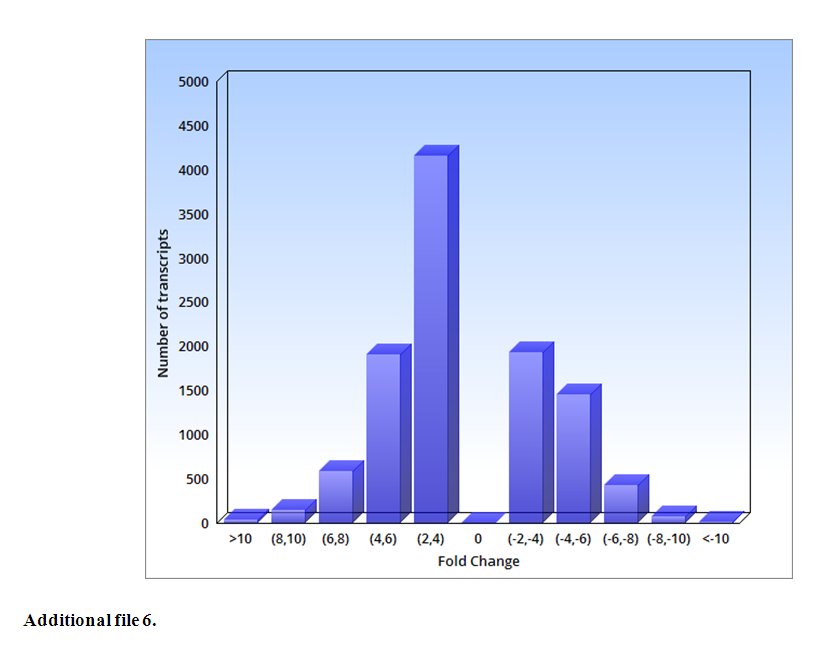

Supplement: Supplementary file 6 — Fold change expression of transcripts in shoot tissue in comparison with root tissue. Differential gene expression was depicted by calculating the RPKMs ratio of each gene in two different independent biological replicates where samples (n = 2). The figure represents the fold change values corresponding to their number of transcripts. Fold change induction along with their RPKM values which shows that maximum number of transcripts are expressed with a fold change induction ranging between 2 to 4 fold for both upregulated and downregulated transcripts. The data represents the mean value of two independent biological replicates with P ≥ 0.05 (significant). (DOCX 75 kb) [file 12864_2017_3865_MOESM6_ESM.docx]

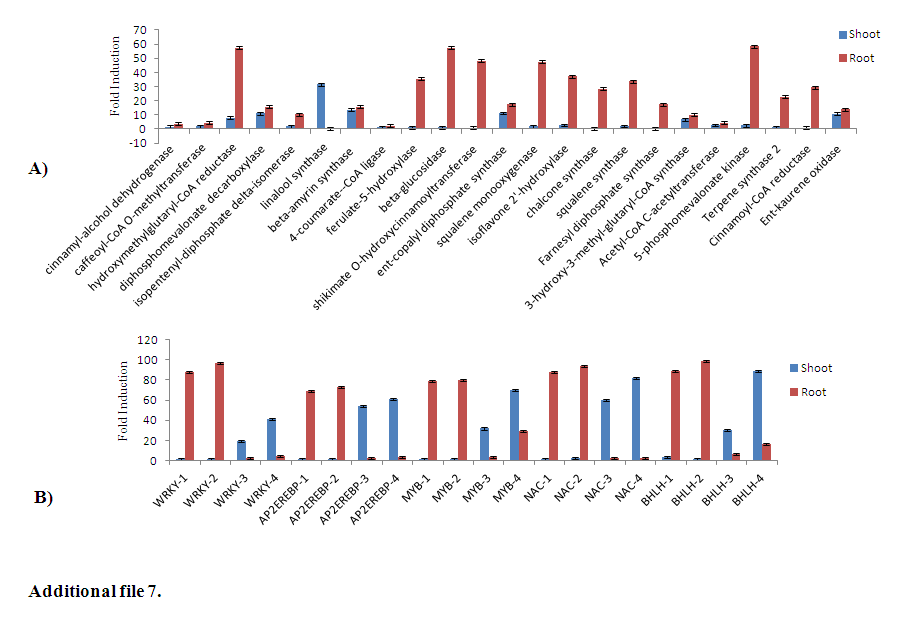

Supplement: Supplementary file 7 — (A) Validation of selected 23 up regulated transcripts in shoot and root by using qRT-PCR analysis. (B) Tissue specific expression of selected transcription factors by using qRT-PCR analysis. All these transcripts were related to secondary metabolite biosynthesis. Actin and ubiquitin was used as the internal reference gene and the relative abundance of each gene transcript in roots and shoot tissue was compared. The data represents three independent biological and experimental replicates performed with standard deviations. (DOCX 69 kb) [file 12864_2017_3865_MOESM7_ESM.docx]

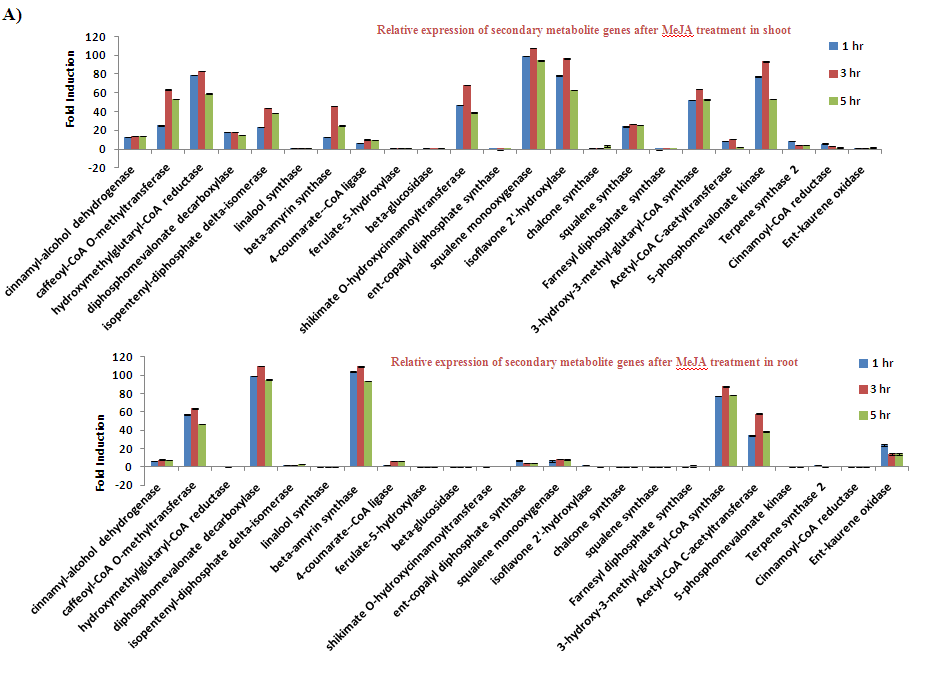


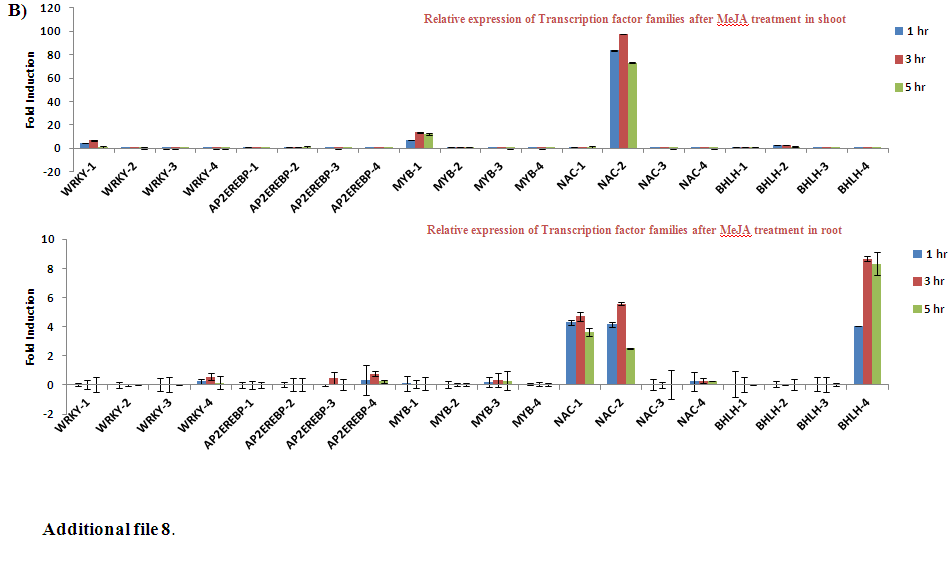


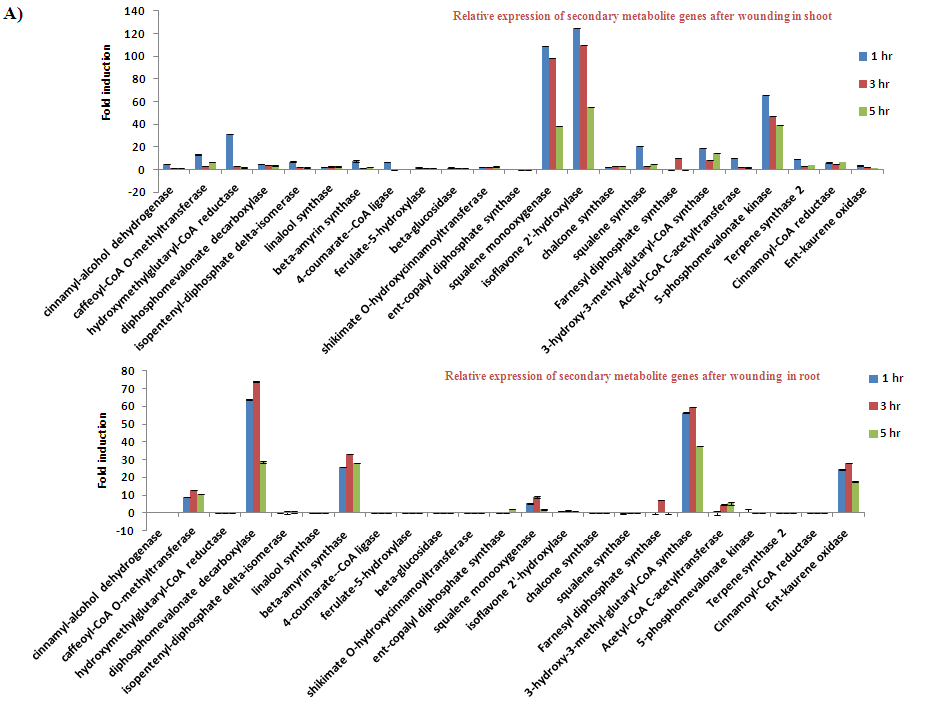


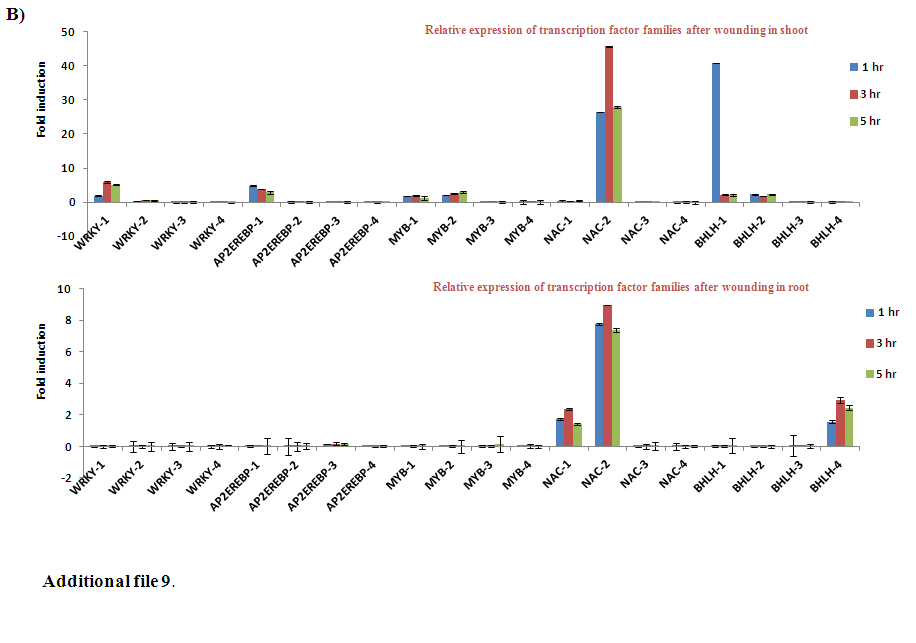

Supplement: Supplementary file 8 — Relative expression levels of secondary metabolism related gene transcripts (A) and Transcription factor families (B) in Bacopa monnieri shoot and root tissues after 1, 3 and 5 h of MeJA treatment. Total RNA was extracted from root and shoot of 1 month old plant and expression level of identified transcript was analyzed by q-RT PCR. Actin and ubiquitin was used as the internal reference gene and the relative abundance of each gene transcript in root and shoot tissue was compared. The data represents three independent biological and experimental replicates performed with standard deviations. (DOCX 269 kb) [file 12864_2017_3865_MOESM8_ESM.docx]

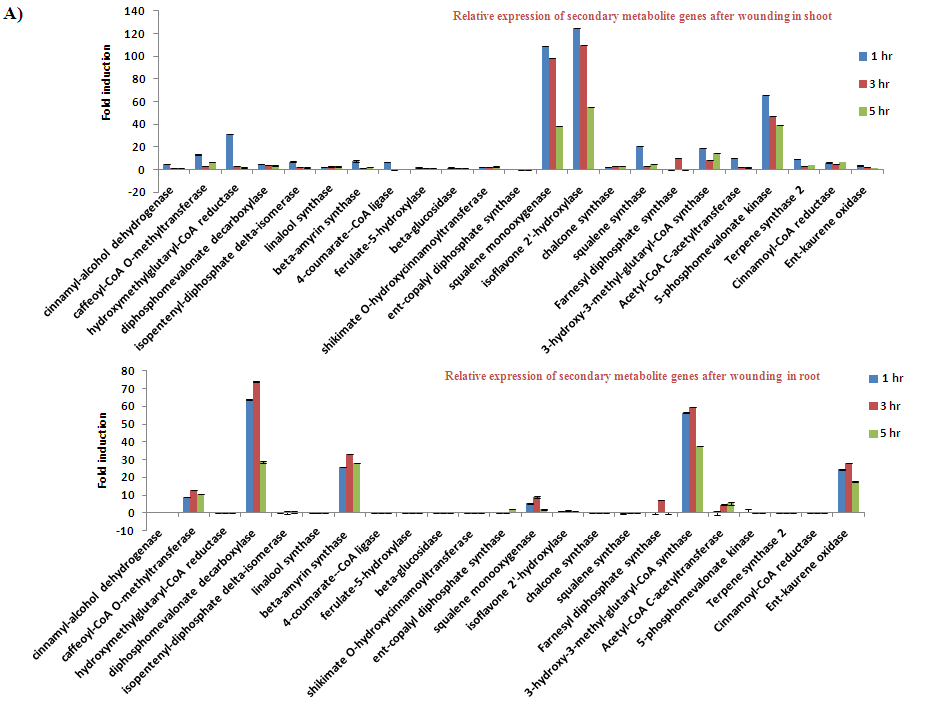


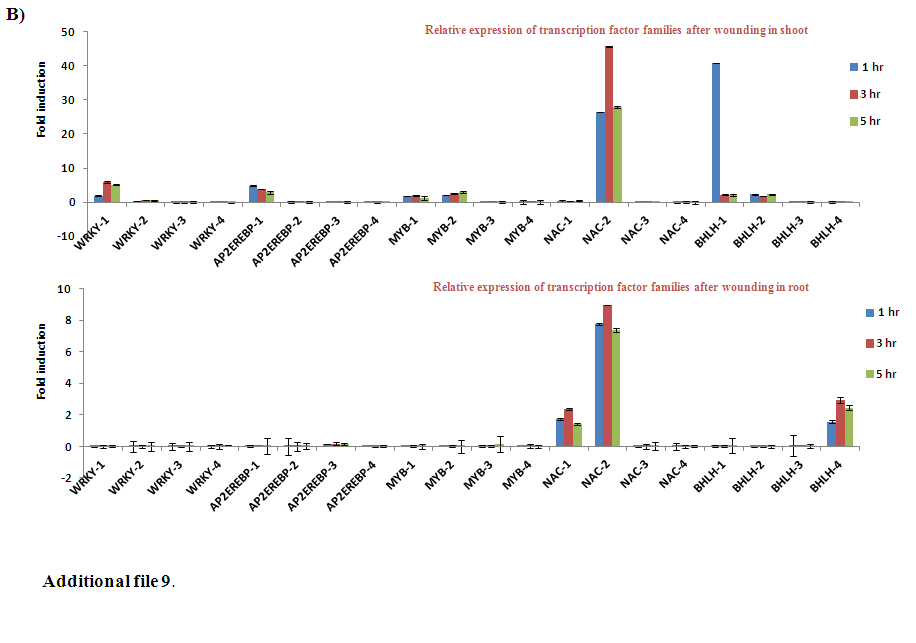

Supplement: Supplementary file 9 — Relative expression levels of secondary metabolism related gene transcripts (A) and Transcription factor families (B) in Bacopa monnieri shoot and root tissues after 1, 3 and 5 h of Wounding. Total RNA was extracted from root and shoot of 1 month old plant and expression level of identified transcript was analyzed by q-RT PCR. Actin and ubiquitin was used as the internal reference gene and the relative abundance of each gene transcript in root and shoot tissue was compared. The data represents three independent biological and experimental replicates performed with standard deviations. (DOCX 140 kb) [file 12864_2017_3865_MOESM9_ESM.docx]
