# Supplementary material for: Comparative transcriptome analysis of shoot and root tissue of Bacopa monnieri identifies potential genes related to triterpenoid saponin biosynthesis
Source: BMC Genomics. 2017 Jun 28;18:490. doi: 10.1186/s12864-017-3865-5 (PMC5490213; doi:10.1186/s12864-017-3865-5)
Supplement: Supplementary file 10 — List of the primers used for selected genes to check their expression using RT-PCR. Parameters used in the velvet and oases assembly. (DOC 125 kb) [file 12864_2017_3865_MOESM10_ESM.doc]

**Additional file 10: Table S1.** List of the primers used for selected genes to check their expression using RT-PCR.

| **Sl No** | | **Target transcripts** |  | **Primer sequences (5′ to 3′)** |
| --- | --- | --- | --- | --- |
|  | | cinnamyl-alcohol dehydrogenase |  | Quantitative RT-PCR |
|  |  |  | F | TCGACAAGCCCGGATTGA |
|  |  |  | R | CGAAAGCCTTAGCGAATTTCAC |
|  | | caffeoyl-CoA O-methyltransferase | F | AGCCGTCATGGCATCTGAAC |
|  |  |  | R | TATGAGACCGGCTTGCCATT |
|  | | hydroxymethylglutaryl-CoA reductase | F | CTTTCCCCTGCCTTCAATCC |
|  |  |  | R | ATGGACGTCATCGGAATCTCA |
| 1. 11 | | diphosphomevalonate decarboxylase | F | ACTTCGATCGCTCGCCAAT |
|  |  |  | R | ACATGCAACTTCTCCCAATCCT |
| 1. 12 | | isopentenyl-diphosphate delta-isomerase | F | CGACCCAATGGAGTAAATTGATC |
|  |  |  | R | TGCCGCTCAAAGGAAGCTA |
| 1. 13 | | linalool synthase | F | AATGGCAAGGTTGGAATCCA |
|  |  |  | R | TGTTGCCAGCTCCAAAAGG |
| 1. 14 | | beta-amyrin synthase | F | CCAAGCATAGCCCATGATGTC |
|  |  |  | R | CGAATCCTGTCCAAGCATGA |
| 1. 15 | | 4-coumarate CoA ligase | F | CGAGATGGTGTGTCCATTCG |
|  |  |  | R | GCCGTTGTCCCTATGAAAGATG |
| 1. 16 | | ferulate-5-hydroxylase | F | GCCCCGGAATGCAACTC |
|  |  |  | R | CCGGTAGATCCCACGTGAAA |
| 1. 17 | | beta-glucosidase | F | AAAGACGTCCCGACTGCTAAAA |
|  |  |  | R | CTGTCGTTGCGGGCGTAT |
| 1. 18 | | shikimate O-hydroxycinnamoyltransferase | F | GGAGCTCCGCAGCCTAATTC |
|  |  |  | R | TGCGTTACCTGCAACACCAA |
| 1. 19 | | ent-copalyl diphosphate synthase | F | CCCGCGAAACAGAAGAATTC |
|  |  |  | R | TCTCAGATTGCACGGTTACGAA |
| 1. 20 | | squalene monooxygenase | F | CGATGGATTGGCCAGGATAA |
|  |  |  | R | TTTGTTGGGATGGTCCTGAAC |
| 1. 21 | | isoflavone 2'-hydroxylase | F | CGCGGATTTCTTCCCTTTTT |
|  |  |  | R | TCCATCTCCCTCCCAACTCTT |
| 1. 22 | | chalcone synthase | F | TCAACCTCCGCCAGGAAAT |
|  |  |  | R | CCCATTCTTCAATCGCCTTCT |
| 1. 23 | | squalene synthase | F | ATGACCTGAGGAATTGCACAAA |
|  |  |  | R | GCATGTCGAAGATTGCATCAA |
| 1. 24 | | Farnesyl diphosphate synthase | F | TTCCTGTTATTGAAGAATCTCCAAAA |
|  |  |  | R | CAAGTACAATTGAGACAAATAGCATATGA |
| 1. 25 | | 3-hydroxy-3-methyl-glutaryl-CoA synthase | F | TGGATGGGAAGAGGGTGGTA |
|  |  |  | R | GACCCTCGTTAAGACGGAGAGA |
| 1. 26 | | Acetyl-CoA C-acetyltransferase | F | ACCCCCAAAAGTGTGACCAA |
|  |  |  | R | CAACGTACATGGTGGAGCTGTATC |
| 1. 28 | | 5-phosphomevalonate kinase | F | AGGTGGCTTTGACGCAGTCT |
|  |  |  | R | ACGTTGAGCGAACTCCACACT |
| 1. 29 | | Terpene synthase 2 | F | CAGGCCCACAGGATGATACC |
|  |  |  | R | AAGCAAGGTGGCATCATAAAGATT |
| 1. 30 | | Cinnamoyl-CoA reductase | F | TGGTTTGAGAATTTGTACGGTTTTT |
|  |  |  | R | GGAGCATTAACTTTACCTTTCATGTG |
| 1. 31 | | Ent-kaurene oxidase | F | CCCAGCTCCGAAAGCCATA |
|  |  |  | F | GAAGAGTGGAAGCCGGAGAGA |
|  | | AP2EREBP-1 | F | TATGCGTCCGAGTAATTTGGTTAC |
|  |  |  | R | ATCATAGCCGGGATGGGATT |
|  | | AP2EREBP-2 | F | GCCATGAGACAGCAGGCTTAT |
|  |  |  | R | AGTGCAAACGAGTCGAGCATAG |
|  | | MYB-1 | F | TCCAAAAGATTACTATCCCAAGAAGAATA |
|  |  |  | R | TAGCCTTTCTGCCCCACAAA |
|  | | MYB-2 | F | TGGAGATCAAACCCCTGGAA |
|  |  |  | R | CAAGCATTAGCCAAGTCGATGA |
|  | | NAC-1 | F | GTGCACGTGATCGGATTCC |
|  |  |  | R | CCACTGCCACCACTTATGGA |
|  | | NAC-2 | F | GTGCACGTGATCGGATTCC |
|  |  |  | R | CCACTGCCACCACTTATGGA |
|  | | BHLH-1 | F | TTATTCTATCTCCTAACTTCTCCTTTCTCA |
|  |  |  | R | GGGTATCTAAGAAGGCTAGGGTTCA |
|  | | BHLH-2 | F | CGGTTGATAACTTCAGCAAGCA |
|  |  |  | R | TCATCTCGCCACCCTTAGGA |
|  | | WRKY-1 | F | GGAAAAAAGGCACCAGAACAAG |
|  |  |  | R | GAATGTGTCACGGGATGTGTTG |
|  | | WRKY-2 | F | TCACAGCTGGCCTTAATTCGT |
|  |  |  | R | GCCGAACATGGCAAGAAGAA |
|  | | AP2EREBP-3 | F | CTGCTGCGACACGTTGGA |
|  |  |  | R | CCGAAAATATTCAACACCATCCTA |
|  | | AP2EREBP-4 | F | GGAAACCTGTCGGGATTGATT |
|  |  |  | R | CGATGGAAACATGGCTAAAGC |
|  | | NAC-3 | F | GAACTCCGGCGGAGTTAGAA |
|  |  |  | R | AGAACGCCTTCCACATGATGT |
|  | | NAC-4 | F | TTCTGCAGGCGAATTCTCCTA |
|  |  |  | R | GTCTCGCCAGCCTTCTAGTGA |
|  | | MYB-3 | F | TGTTATTCTTGGATCTTGATGGCTTA |
|  |  |  | R | GCCGCAATAGCATCAGTTACAG |
|  | | MYB-4 | F | CAGCAGAGCAAGCCCAACTC |
|  |  |  | R | CGGCACGCTACTGTAGAGATCA |
|  | | BHLH-3 | F | ATCTGAGTCGAGCCCCTTCTT |
|  |  |  | R | GTTGCTTGTGTGGGAGTTGGT |
| 1. 32 | | BHLH-4 | F | CGATCGCTCATTCTGGCTTT |
|  |  |  | R | AGCTCGATCCTTAATGGTTGCA |
| 42. | WRKY-3 | | F | TGGCGACACTAATCCGATCTG |
|  |  |  | R | TGGATCGAGAACGAGAGAGATTG |
| 43. | WRKY-4 | | F | TTCCTCCACTGATACCCATCCT |
|  |  |  | R | GGCCAAGATTTCACGGGTTT |
| 44. | Actin | | F | CGATGAGTCAGGTCCAGCAA |
| 45. | Ubiquitin | | F | CCGTGGTGGCCTCTGAAT |
|  |  |  | R | CGGAACAAAAGTCCAGGCATA |
| 46. | Adapter sequence | |  | GATCGGAAGAGCACACGTCTGAACTCCAGTCACXXXXXXATCTCGTATGCCGTCTTCTGCTTG  Where X is index/barcode sequence used for the particular sample. |

**Additional information 1**. Parameters used in the velvet and oases assembly

The default parameters were used for the dynamic and the static filters of Oases-cov_cutoff <floating-point> : removal of low coverage nodes AFTER tour bus or allow the system to infer it (default: 3)

-edgeFractionCutoff <floating-point>: Remove edges which represent less than that fraction of a nodes outgoing flowMust be part of the open interval ]0,1[ (default: 0.01)

-degree_cutoff <integer> : Maximum allowed degree on either end of a contigg to consider it 'unique' (default: 3)

The details on the assembly correction are explained in the section 2.2 of the article Oases: robust de novo RNA-seq assembly across the dynamic range of expression levels.
